# User Control of Personal mHealth Data Using a Mobile Blockchain App: Design Science Perspective

**DOI:** 10.2196/32104

**Published:** 2022-01-20

**Authors:** Arijit Sengupta, Hemang Subramanian

**Affiliations:** 1 Department of Information Systems and Business Analytics College of Business Florida International University Miami, FL United States

**Keywords:** blockchain, mobile apps, mining, HIPAA, personal health data, data privacy preservation, security, accuracy, transaction safety

## Abstract

**Background:**

Integrating pervasive computing with blockchain’s ability to store privacy-protected mobile health (mHealth) data while providing Health Insurance Portability and Accountability Act (HIPAA) compliance is a challenge. Patients use a multitude of devices, apps, and services to collect and store mHealth data. We present the design of an internet of things (IoT)–based configurable blockchain with different mHealth apps on iOS and Android, which collect the same user’s data. We discuss the advantages of using such a blockchain architecture and demonstrate 2 things: the ease with which users can retain full control of their pervasive mHealth data and the ease with which HIPAA compliance can be accomplished by providers who choose to access user data.

**Objective:**

The purpose of this paper is to design, evaluate, and test IoT-based mHealth data using wearable devices and an efficient, configurable blockchain, which has been designed and implemented from the first principles to store such data. The purpose of this paper is also to demonstrate the privacy-preserving and HIPAA-compliant nature of pervasive computing-based personalized health care systems that provide users with total control of their own data.

**Methods:**

This paper followed the methodical design science approach adapted in information systems, wherein we evaluated prior designs, proposed enhancements with a blockchain design pattern published by the same authors, and used the design to support IoT transactions. We prototyped both the blockchain and IoT-based mHealth apps in different devices and tested all use cases that formed the design goals for such a system. Specifically, we validated the design goals for our system using the HIPAA checklist for businesses and proved the compliance of our architecture for mHealth data on pervasive computing devices.

**Results:**

Blockchain-based personalized health care systems provide several advantages over traditional systems. They provide and support extreme privacy protection, provide the ability to share personalized data and delete data upon request, and support the ability to analyze such data.

**Conclusions:**

We conclude that blockchains, specifically the consensus, hasher, storer, miner architecture presented in this paper, with configurable modules and software as a service model, provide many advantages for patients using pervasive devices that store mHealth data on the blockchain. Among them is the ability to store, retrieve, and modify ones generated health care data with a single private key across devices. These data are transparent, stored perennially, and provide patients with privacy and pseudoanonymity, in addition to very strong encryption for data access. Firms and device manufacturers would benefit from such an approach wherein they relinquish user data control while giving users the ability to select and offer their own mHealth data on data marketplaces. We show that such an architecture complies with the stringent requirements of HIPAA for patient data access.

## Introduction

### Background

Data is the new oil.

Clive Humby

This quote by Clive Humby epitomizes the reality of today’s internet-connected society, wherein private firms collect, distribute, store, analyze, and monetize user data. An important but understudied challenge facing the health care industry, users, and service providers of health care apps per se is the dichotomy of standards pertaining to user health care data. On the one hand, there are stringent data requirements such as the Health Insurance Portability and Accountability Act (HIPAA) and the General Data Protection Regulation (GDPR), which assure patient confidentiality, privacy, and security of user data, with most of the burden of data privacy and data security transferred to the provider. On the other hand, there are numerous pervasive devices collecting mobile health (mHealth) data such as height, weight, heart rate, electrocardiograms, sleep patterns, oxygen saturation levels, eye scans, blood pressure parameters, and real-time blood glucose levels with such data stored on devices and later transmitted to private clouds of equipment manufacturers and service providers at scale. Very little oversight or legal protection is provided to users of such devices and mHealth apps to prevent third parties from monetizing such personal data. An illustrious example is when Google recently released a machine learning algorithm that can actively predict a patient’s heart condition by applying deep learning to retinal photographs [1]. In addition, user data are often harvested for research and analysis purposes, which often claim the anonymity of user data.

Although on the periphery, given a choice, >90% of the users surveyed in a recent study chose to keep their data private, and a significant number of them trusted to keep their health data on a blockchain [2]. A challenge facing health care providers, governments, and law enforcement is the cost of enforcing stringent requirements as per the HIPAA [3], GDPR, and the Public Health Emergency Privacy Act. For example, although HIPAA provides requirements for data privacy, the enforcement, monitoring, and penalizing of violators are practical difficulties [4]. The premise of supporting data privacy and access to personal health care data is limited when users sign onto health care apps under *terms and conditions* that prevent unauthorized access without the users’ ability to *share* the data with their provider.

In this paper, we present a novel blockchain-centric approach to pervasive mHealth data by designing and deploying mobile apps that transmit and store data on a configurable blockchain optimized for storing, retrieving, and accessing internet of things (IoT) data. We summarize the blockchain architecture and how it supports IoT transactions using web services. We then develop 2 separate mHealth apps, one on iOS and the other on Android, and demonstrate the novelty of the blockchain-centric pervasive health care apps. We show how users of mHealth apps built atop the blockchain control their data while the blockchain supports access control, privacy, anonymity, and decentralized storage (not in the control of a single firm) [5].

### Objective

To satisfy the challenges of user privacy and data access, combined with the need to maintain high security, speed, and availability of pervasive mHealth data, we propose using a configurable blockchain architecture such as the consensus, hasher, storer, miner (CHASM) architecture [6]. In this paper, we investigate the following research questions:

1. Research question 1: What and how do pervasive mobile apps interface with a configurable blockchain to provide users privacy, security, and control over their mHealth data?
2. Research question 2: How and can pervasive mobile apps adhere to stringent HIPAA compliance?

To our knowledge, this is one of the earliest researches to present a configurable blockchain architecture that combines elements of private and public blockchains and is compatible with the requirements of pervasive mHealth data, which caters to the highest plausible security and privacy requirements as prescribed by HIPAA. Access control is provided at multiple layers, that is, at the wallet layer, at the app layer, and at the blockchain layer. Similarly, prior research has not addressed how such pervasive health care data, which are generated by devices, can be made compatible with IoT blockchain data stores with significant security, high throughput, and expectations of low-synchronization times.

In the following sections, we describe the Design Science Research Methodology (DSRM) and then follow each step in the DSRM to implement a viable solution.

## Methods

### Overview

We used the DSRM, which is commonly used by researchers in information systems and computer science [7]. The main steps in the DSRM are listed in Textbox 1.

Following these principles, we executed the following steps for our solution: first, we defined the research problem and justified both our solution and the value of our solution; second, we defined the design goals for our system as the objectives that we intended to accomplish; third, we designed and implemented our solutions; fourth, we demonstrated the solution with respect to the goals set forth earlier; and finally, we evaluated the solution for accuracy, security, and (potential) costs while documenting the risks.

**Different steps of the Design Science Research Methodology and our approach to the solution.**

- Phase 1
  Step 1: problem definition and importance of solving
  Step 2: identifying objectives of the solution
- Phase 2
  Step 3: design and development of prototype
  Step 4: demonstration of artifact
- Phase 3
  Step 5: evaluation of artifact against requirements
  Step 6: conclusion and communication

### Step 1 of the DSRM: Problem Definition and Importance of Solving

Pervasive devices and apps on such devices that capture individual mHealth data have become popular in everyday use for millions of individuals using wearables, personal health parameter test kits, and medical devices [8]. Such devices and apps have several benefits by positively affecting patient health outcomes through the gamification of health care practices such as increasing the frequency of exercise, monitoring food intake and obesity control, improving communication among patients, and increasing patient motivation by encouraging them to join peer groups of similar individuals [9].

mHealth users generate a significant amount of data through their pervasive devices, which record data such as heart rate, blood cholesterol, and blood pressure. Most often, users own a multitude of devices such as smartwatches, smart apps on phones, smart blood sugar monitors, and smart brainwave readers [2,8,10-12]. Such pervasive devices create large data footprints, and such generated data are usually stored on separate and independent cloud-based servers or network-attached storage. Such databases are centrally administered, and data access is controlled by the firm that manages these data stores after the user’s approval. A key issue with such centrally managed data stores is that users have no control over their data and frequently do not have access to historical data. However, very often, such data can provide valuable insights into user health, and when services such as those demonstrated by Google [1] or by Sleep City [13,14] become more commonplace, it can lead to early diagnosis of medical conditions or provide early warnings about the onset of diseases. Pervasive devices responsible for collecting mHealth data are called edge devices as they have exceedingly small storage capacity and depend on the network to collect and store data [15]. As a result of their resource-lessness and the need to store back-end data elsewhere, blockchains have been shown to provide numerous benefits [16]. However, such benefits are not automatically transferred over pervasive devices and apps that need to be specifically written with security, access, privacy, and performance considerations. Prior health care research on data on health care exchanges, data tamperproofing, and securing health care data have demonstrated the benefits of using blockchains in the context of health care [8].

Several public blockchains are used for building decentralized applications such as those in supply chain management, decentralized finance, and enabling contracts. The blockchain’s advanced features of enabling private-public key cryptography, decentralized consensus algorithms that can be modified, and strong 1-way encryption of data through hashing can address several challenges facing health care data storage [16]. Fang et al [17] discussed the key challenges with current blockchain designs of public, consortium-based, and private blockchains in ensuring that all of the properties required for health care systems (ie, privacy, security, scalability, and immutability of patient health records) cannot be simultaneously available on the same blockchain. This is also known as blockchain trilemma.

The underlying blockchain we developed supports the symmetric encryption of transaction payloads in addition to signing. For this app, we used advanced encryption standards using a *secret key* associated with the user’s private key. A user may associate multiple secret keys for different apps using the same private key. The mHealth data can only be decrypted using the user’s secret key for onward sharing, downloading, and reading. A user can create multiple secret keys for each app, and each app can run on multiple devices to capture information about the same individual. For example, *secret key 1* associated with the user’s ID would be used to capture the user’s heart rate; *secret key 2* associated with the user’s exercise log would be used to capture data on the number of steps a user takes while running or doing exercise; *secret key 3* could be used capture, for example, sleep patterns; and *secret key 4* could be used to log other health care information such as blood pressure, oxygen saturation levels in the blood, and blood sugar. Unique information would be stored in separate wallets or devices and could be deposited or synced up with the blockchain for overall persistent storage.

A feature of HIPAA compliance is providing users with absolute privacy of information and the ability to delete and clean out their data if given a chance. Textbox 2 presents the main requirements of such a software system and why each of these requirements is important to solve.

**A summary of the key requirements and why these are important to solve.**

- Requirement 1: support for continuous data storage outside devices
  Edge devices do not have sufficient storage. As a result, a network-based storage mechanism is needed.
- Requirement 2: diverse types of devices, data, and frequencies that transmit data
  A person’s mobile health care footprint is stored across multiple devices.
- Requirement 3: control access for distinct types of data
  Each device generates a different kind of data and needs separate storage and access control if it were to be accessed by the user.
- Requirement 4: Health Insurance Portability and Accountability Act compliance
  These standards governing health care data are essential to ensure that mobile health data are securely stored and that privacy is ensured.
- Requirement 5: stakeholder incentives for maintaining nodes, mining algorithm, and functionality of the blockchain
  Stakeholders of the system can maintain and control the data on the blockchain. A portion of the revenues from device manufacturers and app sales would be used to fund maintenance, mining, and network maintenance.

### Step 2 of the DSRM: Objectives of the Solution

A blockchain is a decentralized and distributed data store that can address the potential privacy and security concerns related to data access and data standardization while supporting device interoperability and providing users with total control of their own data [10,11]. Blockchain is a secure and immutable transaction ledger distributed on computing devices. Transactions are stored on the ledger through cryptographic validation and linked upward through the network. Owing to decentralization, peers can transact without a third party. Peers access the blockchain through a combination of private–public key cryptography (usually Elliptic Curve Digital Signature Algorithm) and can create and store as much data as they want within the blockchain transaction record. Such transactions are cryptographically signed by the owner of the data using his or her private key and are accessible on the blockchain only by the owner of the public–private key. All stakeholders of data, for example, device manufacturers, device users, marketplace administrators, and health care solution providers, can be permissioned onto the blockchain network [10], or users can explicitly or implicitly share data with other users.

The key advantage of using a blockchain system to track data is that the blockchain can verify and perennially store all created and validated user data. Some of the common advantages of using a blockchain versus a standard pervasive database system are listed in Table 1.

**Table 1:** Advantages of using blockchain for storing mobile health data.

Property of blockchain-based solution	Objectives accomplished for the mHealth app	Standard database or local storage–based apps
Anonymity	A user need not register with his or her personal identifiers and is associated with the data using the private key and public key information.	Access needs to be given by the administrator of the database.
Decentralization	The data is stored on a public infrastructure supported by individual users on a globally distributed network. In our prototype, we tested this with 4 parallel nodes.	Data is centralized and may be controlled by ≥1 administrators.
Transactional safety	Each transaction comprising data is signed with the user’s private key, preventing others from manipulating the transaction.	Administrators are responsible for transactional safety. The database network subsystems are controlled by manufacturers.
Consistency	Irrespective of the type of data being sent on the network, the data is stored on the network as is without modification. In addition, the user may choose to encrypt the data before adding to the transaction payload and also for additional privacy.	The database can modify, alter, and change data when replicated. For example, in distributed databases, the design of the database could ensure that the data is compressed (potentially with loss of information).
Incentivization	Blockchain-based data marketplaces can enable users to be rewarded based on the validation of high-quality data that users can sell to other users.	Databases, by design, do not incentivize anyone. There is a central authority that decides all modes of access.
Perennial storage of data	Public blockchains provide a public space on the distributed ledger to store all kinds of data. With innovative architectures, it is possible for the storage of the data to also exist perennially on the blockchain.	Data can be deleted by administrators or anyone with administrative access.
Privacy preservation	Users who possess their own private key can access their data on the blockchain.	There is no privacy preservation by design. Administrators have all access rights and can give additional access to users.
Pervasive data access across multiple devices, apps, and systems	Users can access an infinite (theoretically) number of wallets, each of which can store a different type of health data. This provides a single-window clearance to all the user’s health data.	Users are limited by the number of access accounts they are provided with by administrators in the system. User accounts are not anonymized either.
Ability to control access to data	The user who has private and public keys can control the data entirely. He or she is the only person that can create the data and access the data (if it is encrypted with his or her key).	Any administrator can control the data.
Ability to prevent access to user data (delete)	Within the blockchain and the pervasive apps, multiple approaches enable individuals to prevent access to the data available on the blockchain. For example, using multi-sig wallets enables data storage to not be accessible after the deletion of one key.	The access is controlled by the database administrator per se.

### Step 3 of the DSRM: Design of the System

#### Overview

**Figure 1:**
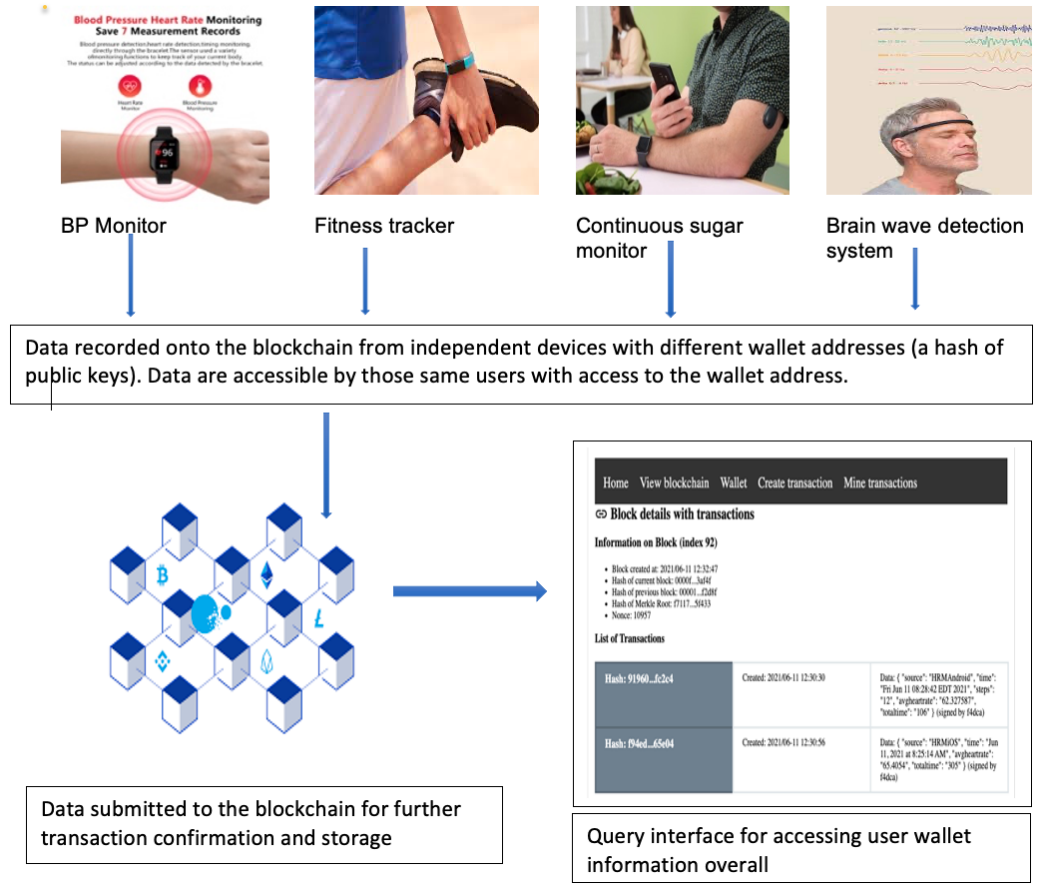
A block diagram showing the process of uploading and managing data with PantherChain. BP: blood pressure.

Figure 1 describes the main use case scenario that motivated our design of the mHealth app. In Figure 1, we see the same individual user using 4 separate devices for monitoring his or her health parameters: blood pressure monitoring device, physical activity (eg, running, jogging, and walking) monitoring, active blood sugar monitoring (eg, for prediabetic or diabetic conditions), and brain wave monitoring. Each device stores and records the individual’s personal mHealth data, which can either individually or collectively be used to make inferences about a person’s health.

#### Use Cases for Storage Retrieval and Messaging

We present different use cases for data storage, data retrieval, and messaging. Apps from different platforms allow users to share data using their private keys to keep the data secure but usable by these apps. These apps can later use whole or part of the user data to provide services, as well as for users themselves to share and control the data provided to the platform.

The use cases are as follows:

1. Use case 1: perform a data collection activity and submit signed data to the blockchain
2. Use case 2: create diverse types of data for the same user from multiple devices using the same private key
3. Use case 3: delink data on the blockchain using private key deactivation (as a blockchain is an immutable structure, data once added to the chain cannot be deleted; however, if the private key is deactivated, that is, delinked from the user, the private components of the data can never be linked back to the user who created it, and any existing encrypted payload data cannot be decrypted in the future or accessed by others)

Other peripheral use cases are as follows:

1. Use case 4: share data across different apps
2. Use case 5: (optionally) sell data in a health care marketplace

**Figure 2:**
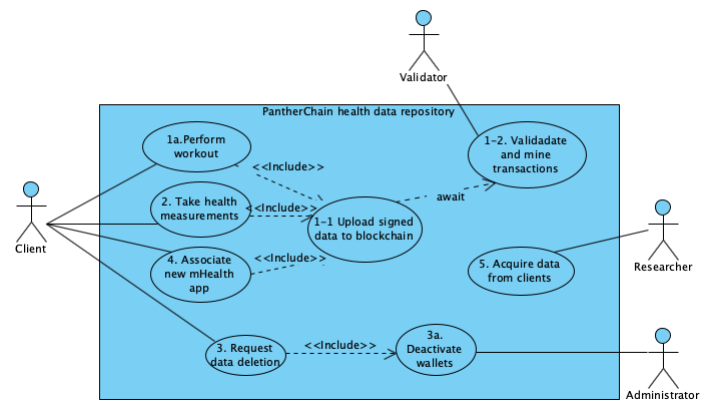
Use case model showing all use cases and dependencies. mHealth: mobile health.

Blockchain design could also incentivize users to create, record, and share authentic, high-quality data and possibly auction the information on health care data marketplaces that access personalized health care data for downstream sale for research. A use case diagram demonstrating all the above use cases and their dependencies is shown in Figure 2.

We extend prior research on configurable blockchain patterns and specifically use the CHASM blockchain pattern and the corresponding instantiated *PantherChain* design to accomplish storage, retrieval, and market functionality for health care data [6]. In the following sections, we describe the design of the PantherChain system and document features of the PantherChain blockchain system used to implement key use cases for pervasive IoT-based mHealth data. The advantage of using the CHASM design pattern is that it is flexible, and each underlying component can be altered to suit the performance, speed, and storage needs of the overall IoT system. Sengupta and Subramanian [6] demonstrated the design and performance functionalities of 4 different blockchains by altering the software components. We present the architecture of such a system and illustrate the benefits of such an implementation in the following sections, which include high performance and tunability of parameters such as difficulty used in mining, especially when IoT devices and high throughput are required.

#### The CHASM Design Pattern and PantherChain Implementation

We used the CHASM software design pattern and the implementation of this pattern in Java that we call *PantherChain* [6]. Sengupta and Subramanian [6] described the CHASM in greater detail. However, they did not configure PantherChain or CHASM to support IoT through web service–based application programming interfaces (APIs) for smart contracts. In this implementation, we adapted CHASM’s configurable architecture and implemented an IoT-based extension to CHASM in the form of a representational state transfer (REST) API layer that accepts requests from devices and supports high performance and throughput.

**Figure 3:**
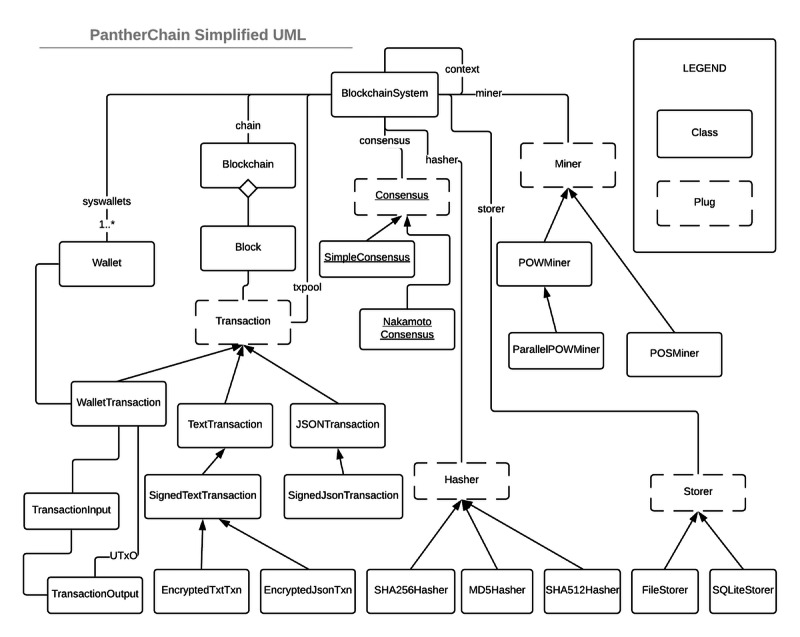
Unified Modeling Language (UML) class diagram of the PantherChain blockchain.

In Figure 3, we show the CHASM design pattern by illustrating the classes and their interactions. The CHASM pattern is an extension of the popular context object design pattern [18], a core Java 2 Platform Enterprise Edition pattern that allows different classes to share a single controlling context (interface). In our implementation, the BlockchainSystem class references abstract class implementations of the 4 CHASM components: consensus, hasher, storer, and miner. Henceforth, we will refer to these concepts, implemented using abstract classes, as *plugs*.

In PantherChain, we provided different implementations for each plug. The *hasher* plug has 3 implementations: MD5 (Message Digest Version 5) [19] implemented as MD5Hasher, SHA256 (Secure Hash Algorithm) [20] implemented as SHA256Hasher, and SHA512 [20] implemented as SHA512Hasher. The storer plug has 2 implementations: a serialized JSON file implementation (FileStorer) and a relational implementation using SQLite (SQLiteStorer). The *miner* plug was implemented as a *proof-of-work* (POW) miner with flexible levels of difficulty, similar to Bitcoin [21]. We included 2 implementations of POW: a single-threaded POWMiner and a multi-threaded parallel version (ParallelPOWMiner). Finally, the consensus plug was implemented using a distributed file-sharing mechanism as a proof of concept to demonstrate a distributed Nakamoto [21] consensus, as described by Nakamoto [21] (SimpleConsensus). PantherChain can be extended by replacing the *plugs* (implemented as abstract Java classes). Each plug provides specific abstract methods that are interfaces to the rest of the blockchain system. Finally, PantherChain can use any implementation of transactions and can be used for different types of transactions (eg, text-based or cryptocurrency-based) within the same instance. In PantherChain, both text-based and JSON-based transactions can be signed and encrypted. Interestingly, as the payloads of encrypted JSON transactions are no longer well-formed JSON, the encrypted JSON transaction class is a subclass of signed text transactions (Figure 3).

### Step 4 of the DSRM: Implementation of the IoT Interface on the Blockchain

#### Overview

Although smart contract [22] virtual machines are not implemented within the CHASM pattern, we piggybacked on the Java Virtual Machine combined with an easy-to-use web service–based software as a service system that operates on each PantherChain node as the IoT interface. The current implementation of PantherChain includes a Java 2 Platform Enterprise Edition Servlet–based REST API that allows an external app to use all components of PantherChain. Some of the end points in the PantherChain REST API are as follows: *APIGetKey* (to retrieve an existing wallet or create a new public or private key wallet), APIAddJson (to add a transaction containing JSON data signed with a key), and *APISearchUid* (to search PantherChain based on a unique identification).

To implement a smart contract, the app can make an API connection to a PantherChain node and provide the client with complete app integration. This method allows app developers to use a blockchain in the back end and develop their app logic in the language or platform of their choice. We developed and tested each of our use cases listed above on PantherChain to demonstrate the capabilities of the system and validate it with user-based tests and HIPAA compliance guidelines. We also set forth future research directions for such a model by illustrating incentivization models based on third-party validation of data accuracy, marketplaces for data, etc. The PantherChain implementation also includes the demonstration of Bitcoin-like cryptocurrency (PantherCoin) atop the existing system.

#### Invocation of the IoT mHealth Data Interface

Integrating PantherChain into an mHealth system is a 2-step process: (1) initializing or retrieving a user wallet (using APIGetKey) and (2) submitting a transaction to the pool using APIAddJson. In our tests, we developed 2 apps, one for an Android watch and the other for an Apple watch, each of which can capture some health data from the sensors, build a JSON data packet, and send it to the API back end. Multimedia Appendices 1 and 2 show sample Android (Java) and iOS (Swift) codes for performing this action.

## Results

### Step 5 of the DSRM: Demonstration of the Mobile Data Health Care iOS and Android Apps

#### Overview

To provide a proof of concept of the process, we developed 2 watch apps: one based on Android and the other based on iOS WatchKit. Both were simple apps that were designed to run on a watch. We programmed the Android app to track steps and heart rate data when a *start* button is tapped and submit a signed package containing the average heart rate and total steps taken when the *stop* button is tapped. The iOS WatchKit app worked in the same way, except that, given the iOS watch we used did not have a direct internet connection, the data were uploaded by an iPhone companion app that retrieves the data from the Apple watch and submits a similar signed package to the blockchain. The user can set up both apps with the same wallet (private-public key pair) so that the data from all the devices can be linked to the same user. Alternatively, the user could use a meta-wallet such as metaMask [23] or the Exodus [24] wallet to generate a master public-private key to store independent wallets within the meta-wallet. Overall, irrespective of the approach chosen, all the user’s data and confidential information are captured and stored only by a single user. In the following sections, we provide some illustrations of the apps we designed, thus demonstrating the various use cases above.

#### Implementation and Validation of Use Case 1

In this use case, the personal health care data of users are stored and accessible on the blockchain using a query interface using the private key by a single user.

**Figure 4:**
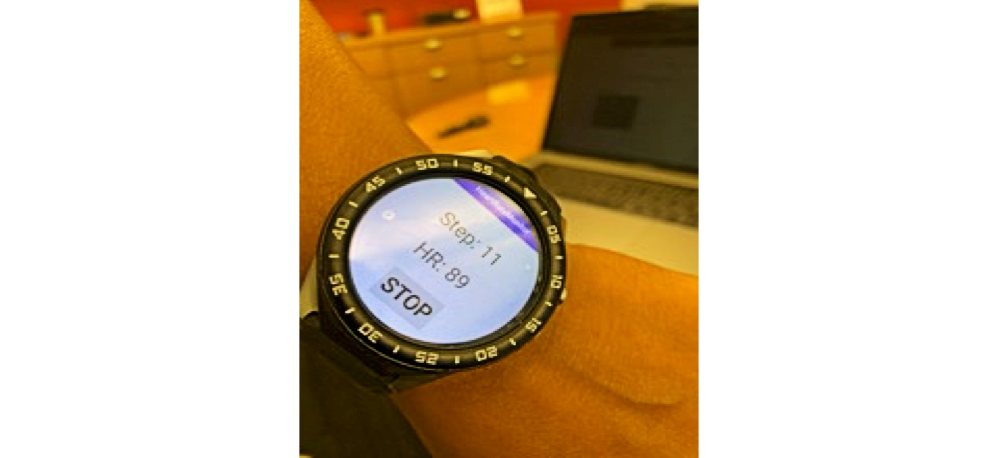
Android implementation of our app running on an Android smartwatch.

The first basic use case involves a user performing a basic workout using an Android watch. Figure 4 demonstrates the Android app running and collecting data while the workout is in progress. The app may enable the encryption of the payload for additional security of the data. We will discuss the encryption process further in the *Implementation and Validation of Use Case 3* section. When the workout is stopped, the watch (or the controlling phone if the watch does not have direct internet access) can submit the workout as a new transaction to the blockchain after encrypting it with a secret key (if enabled at the app level).

#### Implementation and Validation of Use Case 2

In this use case, personal health care data of diverse types with multiple devices and meta-information are stored on the blockchain.

**Figure 5:**
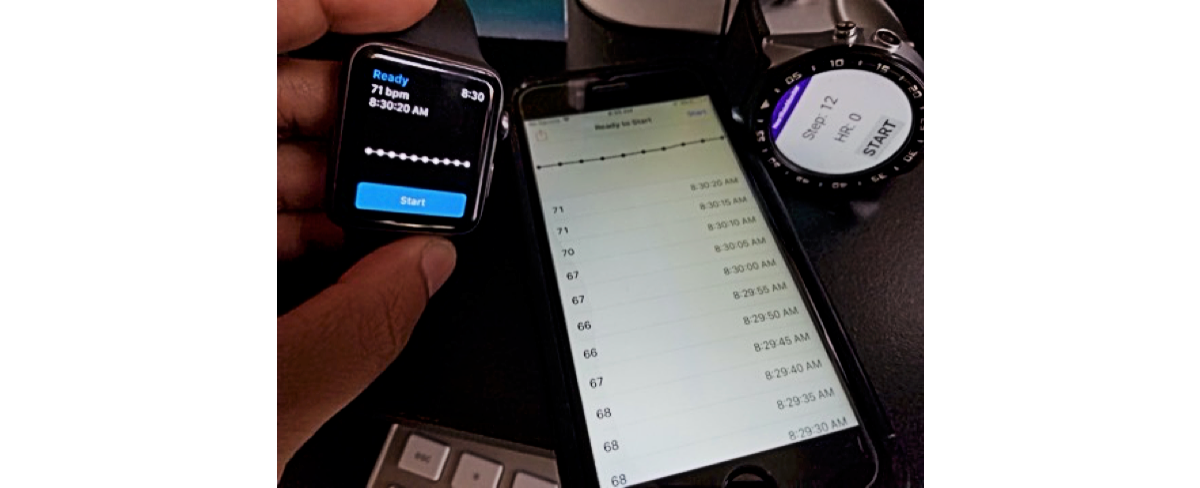
Different devices collecting health care data.

Although the first use case is the most basic method of tracking mHealth data, similar processes exist in most health monitoring platforms today. The motivation for use case 2 comes from the limitation of the ability to share and aggregate data from different mHealth systems into a single platform. Currently, users do not have the option of using different devices with differing capabilities of collecting health data and reviewing and aggregating all data in a single coherent manner. Our app solves this issue by allowing different apps to collect mHealth data to submit data packages signed with the same wallet to be submitted to the blockchain. Figure 5 shows the 2 apps in iOS WatchKit (left) along with its companion iPhone app (center) and the Android watch app (right), collecting diverse types of data for submission to the same blockchain.

Figure 5 illustrates 2 different devices (an iOS device and an Android device) collecting health care data from the same individual and submitting the data to the blockchain. The user of the app, identified by his private-public key combinations, can access the data within his wallet from a web app (as shown in Figure 1). Such data collected about the user from multiple devices can be used to create a health intelligence dashboard and can also be traded (shared) with data marketplaces.

#### Implementation and Validation of Use Case 3

In this use case, personal health care data are deleted based on the private key of the individual after deboarding from the system.

To provide HIPAA compliance, users need to have the ability to delete personal health data from any associated system. As a blockchain is immutable, data cannot be tampered with once it is mined and added to the blockchain (but it can certainly be deleted from the transaction pool before it is mined). However, in our mHealth implementation, HIPAA compliance can be achieved by deactivating the user wallet, which delinks any mined block from the user. If the user chooses to encrypt the data payloads for the transactions, the encrypted components cannot be decrypted without the user’s secret key. However, the blockchain integrity can still be maintained with blocks containing the data, although no association with a specific user can be made without the wallet.

We highlight some other potential approaches for privacy implementations in the following sections.

#### Implementation and Validation of Multi-key Wallets

**Figure 6:**
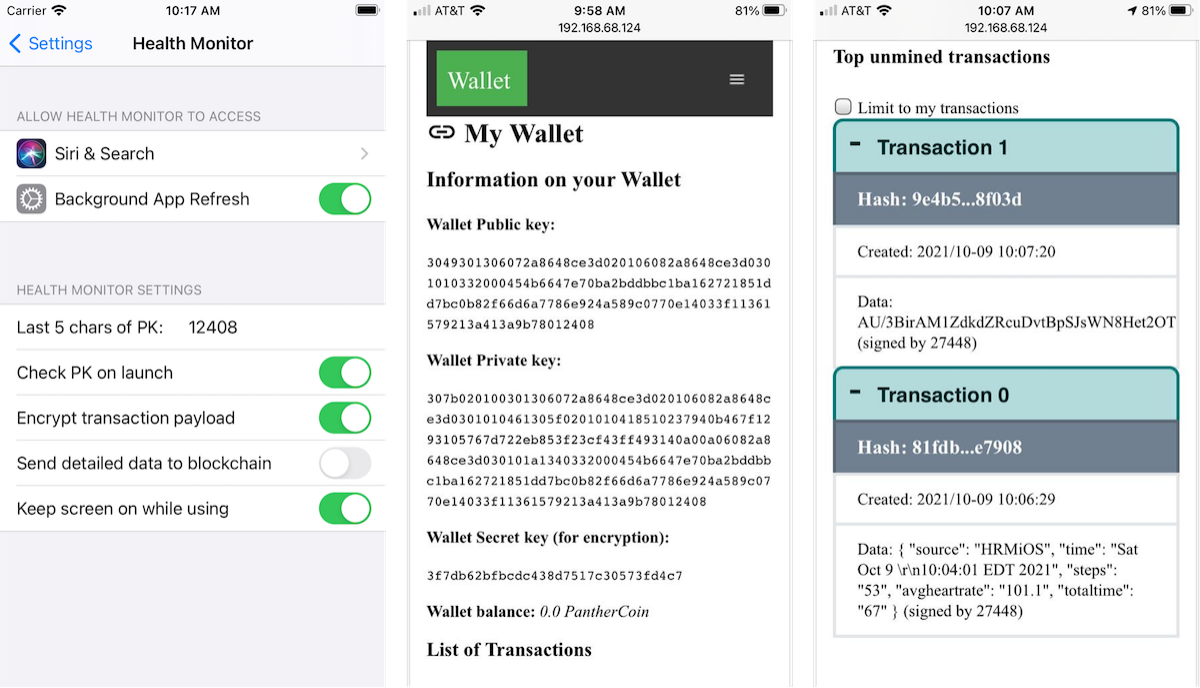
Implementation of encrypted transaction payloads in PantherChain and health monitor app.

A wallet in our app can be associated with multiple secret keys for encryption. Deactivation of any key associated with the wallet renders the transactions encrypted with that key inactive. In our implementation, a wallet included a public and private key pair for signing and verification and several advanced encryption standard secret keys for encrypting payloads. Figure 6 demonstrates (1) enabling the user of the mobile app to encrypt transaction payloads; (2) a wallet with a public key, private key, and a single secret key; and (3) a simple signed transaction and a transaction from the same user with an encrypted payload.

#### App Access Restriction

Privacy compliance can be implemented at the app level, where the user requests the deletion of an account, leading to the removal of the user’s wallet from the blockchain. Once the user deboards the system, if the private keys are removed from the personal devices of the users, the data can never be reconnected to the user level.

#### Implementation and Validation of Use Case 4

In this use case, personal health care data with different types of data are aggregated into a blockchain app.

Once data from different apps are uploaded into the blockchain, a third-party verification method can validate the authenticity of the data (this process may differ from blockchain to blockchain but will involve some form of mining strategy). In this proof-of-concept app, we used a POW mining technique (similar to that of Bitcoin but using a lower difficulty level) to combine validated transactions into mined blocks. These validated blocks can be used by apps to aggregate data from every app that uses the same wallet to be associated with the same user. The mining module in the CHASM pattern in PantherChain is implemented as an interface [6]; hence, it can be replaced with other algorithms such as Proof-of-Stake, which can provide deterministic, time-bound confirmations for transactions logged into blocks [25].

#### Governance Approaches for PantherChain

One of the largest challenges with IoT-based blockchains is related to who governs the blockchain. Governance functions for the blockchain include the ability to control the functionality of the blockchain [26]. This could include altering algorithms for consensus, hashing, storage, and mining. In addition, the blockchain’s future functionality enhancements, including the ability to support different apps on top, could be subject to governance. Governance protocols that can be considered for the blockchain can be of many types. Table 2 summarizes the key governance mechanisms of the token.

On the basis of our analysis of an mHealth-based app stack, which interfaces IoT with the blockchain, the *consortium-based* blockchain works best. If a consortium of device manufacturers, app developers, and users cannot be formed, creating a decentralized autonomous organization facilitated by PantherCoin, the inbuilt token of the PantherChain blockchain, will enable such a mechanism. However, the risks of such an approach are greater than having an equitable consortium of stakeholders who invest and control the blockchain and its future enhancements and can ensure decentralization. For a system such as ours that deals with health care, it is recommended that governance not rely on tokenomics; rather, the decentralized functionality of the blockchain should be used overall.

**Table 2:** Governance mechanisms recommended for the blockchain.

Type of governance	Description	Benefits	Challenges
Consortium-based governance, which is a user group comprising device manufacturers, users, and health app writers; prior apps have included IoT^a^-based power networks [25]	A group of firms, trusts, and user groups pool in resources and administer the whole network of nodes. Membership to the consortium would be rule-based and inclusive. Members could include private organizations, departments of health, and nongovernmental organizations, which are responsible for running and maintaining this structure.	Such a design automatically ensures that the members of the consortium are invested in governance. Adoption is almost instantaneous as the network is already seeded by the consortium. As CHASM^b^ is a configurable blockchain, underlying algorithms and methods can easily be altered, as quorum among consortium members is easy to accomplish.	Typical issues of collusion, exclusion and nonadherence to the rules of governance could slow adoption or reduce the number of users. The consensus algorithm (and the miner plug) could be moved to proof of stake from proof of work, which is a change at the underlying level. However, with proof-of-work mining, the mining algorithm could be operated by the consortium itself.
DAO^c^ [26]	Governance is typically decided by the voting rights of users who own crypto-tokens generated by the platforms and which are purchased in exchange for fiat currency. Voting rights are usually proportional to the share of tokens owned by those who choose to govern.	Everyone ideally has a chance to participate in governance. It could potentially lead to faster adoption as governance is also incentivized.	Governance is usually skewed among those token holders who hold the largest number of tokens. This could result in a dysfunctional blockchain if the tokenomics do not reward users appropriately. There are risks of rug pulls in the market.
Public blockchain [27]	This is similar to the creation of any large public blockchain. All users have equal rights on the network, and peer nodes handle the traffic.	Public blockchains foster trust among individuals for easier adoption. Public blockchains are useful for internal purposes as well as external purposes.	It involves extremely slow adoption. There are no special incentives to users and node runners. Network effects will become extremely difficult to run and maintain. Time taken to modify and roll out changes will disincentivize device manufacturers and users.
Private blockchain controlled by device manufacturers	This blockchain is similar to hosting a set of nodes and controlling their data privately on the network.	It is easy to set up as private investors are involved. Changes to the underlying blockchain are all dictated by individuals on the network overall.	It is controlled by private actors. Decentralization of governance, modifications, and data is difficult to accomplish overall.

^a^IoT: internet of things.

^b^CHASM: consensus, hasher, storer, miner.

^c^DAO: decentralized autonomous organization.

#### Cost of Setting up a Blockchain

There are many types of costs associated with setting up such a blockchain, such as the costs for programming, the cost of hardware setup, the costs for setting up a network, and the cost of app development and integration with device manufacturers. Although this paper will not be sufficient to cover all these costs because of the detailed cost analysis needed, we have documented the cost of hosting our prototype. We hope that designers and developers will be able to interpret the hosting costs from these figures and find these costs significantly lower than those of the public blockchains or other consortium-based blockchains, which require more specialized knowledge and higher bandwidth equipment such as application-specific integrated circuit (ASIC)–based processors for mining.

Our prototype instance was configured and set up on a cloud virtual machine with 1 TB disk space, 128 GB RAM, and Linux with 2 core Intel processors. The cost of setting up 1 instance of the blockchain was US $300 for 3 years. Similarly, nodes could be set up at US $1200 for 3 years. This is for unlimited incoming and outgoing network traffic. We used a common high-performance computing infrastructure cloud available to us from the university. A similar hosting arrangement could be applied to the cloud environments (such as Amazon Web Services, Google, Microsoft, and Heroku) or even with blockchain-based distributed computing clouds such as FileCoin, Storj, or InterPlanetary File System–based systems, which could cost the same amount. On the basis of the number of consortium members and the number of device manufacturers or devices that store data on the cloud, use could also increase or decrease as time goes by. These systems are flexible enough and can accommodate the need to store only the most recent data (and partial or full replication of data could be supported). As the CHASM architecture is flexible, future versions could include sharding of the data store and other performance enhancements that are typical of large-scale data stores. A full discussion of distributed systems architecture would be out of the scope of this paper as we focus on the privacy and user control of data using an IoT-optimized data store.

#### Performance Statistics for the PantherChain System

Although the performance of blockchain systems has been subject to much debate in the literature, it must be noted that performance is measured in terms of the number of transactions that the blockchain can handle per minute overall. For example, the VISA network supports 1700 transactions per second, which indicates a submillisecond access time for any web service request on the network. The Bitcoin and Ethereum networks are much less performant, and these systems have not been benchmarked from an app standpoint.

Our approach to testing the performance of the PantherChain is based on the caveat that the performance of any system can be assessed by measuring the overall times taken to access different components of the system. In our case, owing to the architecture of a distributed and decentralized computing system, we could measure the overall performance of our system in terms of the time taken to access the services of the blockchain. For example, the time taken to access the front end of PantherChain and the time taken to access the REST APIs are discussed above. As we use the Apache Tomcat web server, using the software as a service model, several performance benchmarks of the stack exist. Notably, the Center for Internet Security benchmarks for Windows and Linux operating systems and several processor-related benchmarks already exist [27]. Similarly, Apache Tomcat and Java performance benchmarks for a variety of hardware and software, including combinations of Java Development Kit and Java Virtual Machine implementations, have already been performed [28]. For the performance testing of different configurations of blockchains, please refer to the study by Sengupta and Subramanian [6].

**Figure 7:**
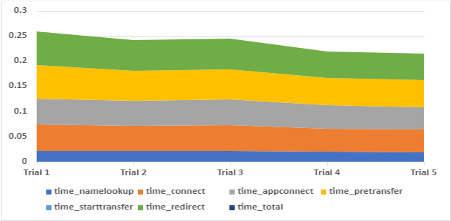
Mean performance of application programming interface response times from PantherChain implementation.

We benchmarked the standard typical use cases of the front end of the PantherChain system and the basic API for obtaining the wallet key after generating it. In Figure 7, we plot the means of the API response times on 5 dimensions commonly used to measure the performance of the getKey and uid APIs and the PantherChain user interface.

### Validation of the Artifact

We provide a complete validation of our process, against the requirements, including the HIPAA compliance checklist for mobile devices [29], in Textbox 3.

**Confirmation of the achievements of the design goals for the system.**

**Health Insurance Portability and Accountability Act guidelines and how our internet of things mobile health app and blockchain design support the functionalities**

- Use a password or other user authentication
  Users use a combination of private and public keys that they must access for personal mobile health data.
- Install and enable encryption
  All data are encrypted at multiple levels. At the storage level, users could choose to encrypt and send data using their public key (or a separate key), and their wallet software could enable access to these data later.
- Install and activate remote wiping or remote disabling
  As discussed, the deletion system for personalized mobile health data can be deleted at the app level and the individual level.
- Disable and do not install or use file-sharing apps
  File or data sharing is not enabled at the app layer, although the blockchain resides on a decentralized network. The access to data is provided only to those with the private and corresponding public key (wallet) and not to anyone else.
- Install and enable a firewall
  The strong encryption provided by the network and the iOS or Android operating systems for mobile health data provides necessary protections. Overall, if firms choose to provide an operating system–level firewall for other types of data from devices such as blood pressure monitors, they can do the needful.
- Install and enable security software
  This is outside the scope of our app, although we believe that pervasive devices are compliant with security.
- Keep your security software up to date
  Blockchain software, when updated, will reflect the same at the app layer, and users will be able to directly contact the apps.
- Research mobile apps before downloading
  This is a user characteristic, and users who work on data should be cautious while downloading apps. Either way, without private and public keys, users will not be able to move data onto the blockchain.
- Maintain physical control
  Pervasive health care data are strongly controlled by the user’s ability to protect their public and private keys and, therefore, control access.
- Use adequate security to send or receive health information over public Wi-Fi networks
  Data can be encrypted end to end using HTTPS protocols to allow only secure apps from devices to communicate with the blockchain’s web service layer.
- Delete all stored health information before discarding or reusing the mobile device
  Once a request is made to delete a particular private key’s data by the user, the user can deactivate all the data from the app by preventing access to it forever. Whenever a mobile device needs to be reused, it is up to the user to remove his or her private and public keys from the device.

## Discussion

### Evaluation of the Prototype

In this study, we developed a complete blockchain-based mHealth data collection system using PantherChain, an implementation of the CHASM-based blockchain framework. We developed mobile apps capable of running on smartwatches to collect personal health data, which are signed with the user’s private keys, and demonstrated that this process could be implemented in high-performance apps. We tested our results with a proof of concept, as explained in detail in previous sections, using both an iOS and Android mobile app and the blockchain. We can clearly show evidence for access to diverse types of health information [6] by the same user.

Further results demonstrating the performance of our app in the context of health parameters and different operating system versions are documented in the following paragraphs.

**Figure 8:**
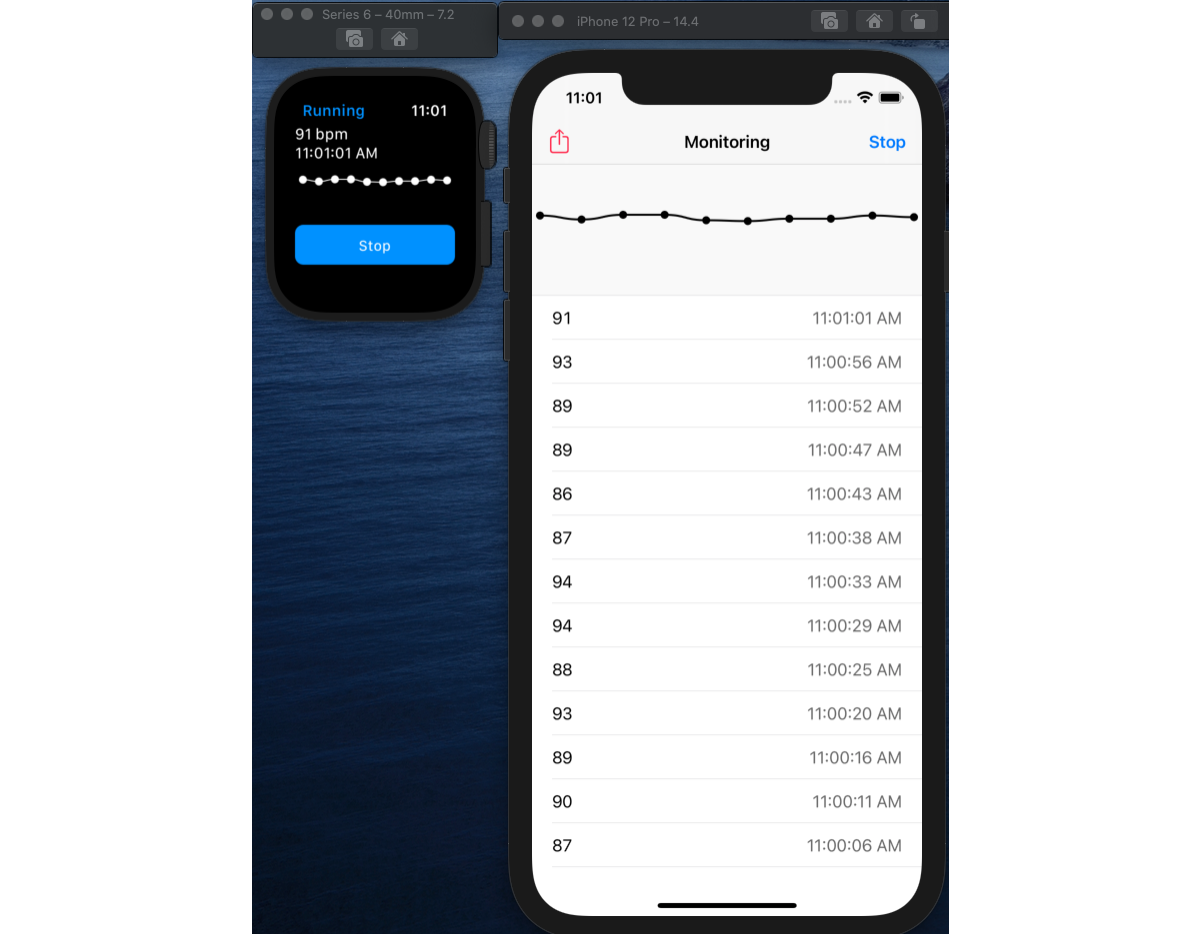
Depicting the simulators showing Android apps sending data to the blockchain.

We collected performance data by running our proof-of-concept apps on different devices and simulators. The proof-of-concept iOS WatchKit app was tested on the following platforms:

1. iPhone 12 Pro simulator running iOS 14.4 paired with Apple Watch Series 6 40 mm simulator running WatchOS 7.2 (Figure 8).
2. iPhone 6 device running iOS 12.5.4 paired with Apple Watch (original) running WatchOS 4.3.2 (Figure 5)
3. iPhone 12 device running iOS 14.6 paired with Apple Watch Series 3 running WatchOS 7.3.2

Our proof-of-concept Android watch app was tested on the following platforms:

1. Android Watch device ZGPAX S99c running Android 5.1 Lollipop (Figure 4)
2. Android Watch simulator (not reported in results because of unavailability of health simulators) running Wear OS 9.0 (Android Pie)

**Figure 9:**
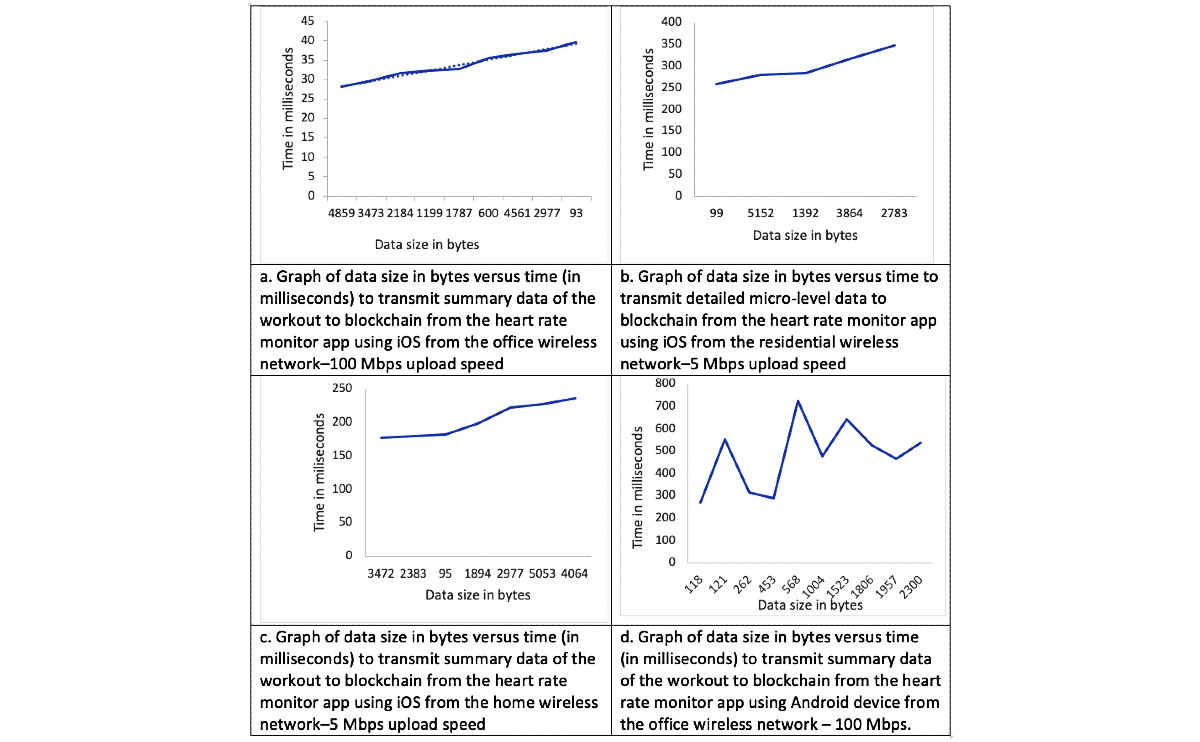
Blockchain submission modes for internet of things performance testing for different network and device combinations for the Heart Rate Monitor (HRM) app.

The host platform for the 2 simulators we used was a MacBook Pro 2018 (Apple Inc, 2.6 GHz Core i7, 32 GB RAM) running macOS Catalina. The work network was a wired 1 Gbps network, and the home network had a maximum upload speed of 5 Mbps. Note that the Android watch device we used had only a 2.4 GHz wireless adapter (which is typical of most low-cost, high-performance IoT platforms, as was available during the writing of this paper). We tested on some old hardware to ensure that our apps could run with adequate performance on low-performance IoT devices. After running our apps, which were configured to send summary as well as detailed workout data, and varying the time of workouts, we noticed that the performance of submitting workout data to PantherChain primarily depended on network latency, network throughput, and HTTP protocol handshake time. The round-trip time from device to PantherChain was observed to be as low as 28 milliseconds (simulator in 1 Gbps network) to a maximum of 724 milliseconds (Android 5.1 device on a 2.4 GHz home wireless network). We collected data for workouts ranging from 1-minute to 30-minute durations, and even with detailed data uploads, the performance was reasonable and as expected for any network-connected device. We plot the graphs of the time taken to submit the data to the blockchain in milliseconds (y-axis) versus the size of the data (in bytes) payload on the corresponding device for the 4 configurations in Figure 9.

The 4 configurations correspond to using the heart rate monitor app running in 4 different modes on different network configurations, as shown in Table 3.

The results demonstrate that our platform and process for collecting and submitting health data to a blockchain does not degrade user experience in terms of performance. Please note that the performance times reported are only based on submission to the blockchain and do not include mining performance, which is not necessary unless the user wants to have the data committed to the blockchain for potential marketplace use. Mining performance comparisons for various difficulty levels (by varying the mining algorithm) of PantherChain are available from the study by Sengupta and Subramanian [6].

**Table 3:** The 4 configurations and the type of data collected.

Device	Type of data collected	Network configuration
iOS	Summary	Work; wired
iOS	Summary	Home; wireless
iOS	Detail	Home; wireless
Android	Detail	Home; wireless

### Comparison With Prior Work

Although some prior works have provided a methodology for achieving tamper resistance and privacy in storing mHealth data in blockchain (eg, Ichikawa et al [8]), our implementation takes it further in a full blockchain implementation using actual mHealth data apps. Fang et al [17] suggested using a permissioned private blockchain to accomplish some of the GDPR capabilities to enable users to control their data. Scholars have developed and demonstrated apps for blockchain in the areas of health care exchanges, mobile data exchanges, and centralized health record storage. Such work has mostly been with enterprise health care systems such as electronic health care records and whose primary use case for the blockchain is for decentralizing data storage [30-34]. Although such apps are built and deployed on either Hyperledger or Ethereum frameworks, they are not necessarily tested for IoT compliance, which is at the other end of the performance, data size, and speed spectrum. Griggs et al [35] discussed that with public blockchains, no user data should be stored within smart contracts as HIPAA compliance indicates that such data are not private and are accessible to all. Our configurable blockchain design considers this limitation and solves this problem by encrypting the data and allowing data access only through wallets that are owned by users. Similarly, we can also configure the entire blockchain on a private network and not on public blockchain infrastructure, which prevents public viewing of such data.

Several prior papers have cited and used Ethereum as an underpinning technology for smart contract–based blockchain designs. However, with Ethereum as the blockchain, on-chain storage is expensive, and for IoT apps such as edge devices, which require storage of data on the chain for fast retrieval or health care analytics, Ethereum may not be best suited as a blockchain. A recent estimate for the Ethereum blockchain showed that storing 1 MB of data on chain would cost approximately US $76,000 [36]. As a result, on-chain storage on Ethereum is not recommended for current apps. Storing off chain will also be significantly inefficient because of the gas costs on Ethereum for writing a transaction and operating a smart contract, which today costs approximately US 25$ to US 60$ per transaction, which would be more expensive than some of the low-end smartwatches in the market.

Another limitation of prior work is that with mHealth data and pervasive computing, the need for data platforms to operate in the context of multi-platform data sources has not been tested. Pervasive (edge) devices that need to transmit data frequently to back-end data stores present specific network transmission time (performance), security, and privacy challenges. The web services architecture (CHASM) that we deploy is platform independent, and we demonstrate using Android and iOS apps with different network configurations, all communicating with the same blockchain web service for 1 individual user. Overall, we believe that our approach is a novel method for designing and developing and demonstrating the functionalities of a HIPAA mobile data–compliant system that comprises pervasive mobile data apps on multiple devices and a custom configurable blockchain that is platform independent and performant with respect to IoT characteristics. We conclude our research by stating the benefits of using such a configurable blockchain architecture (CHASM) with a web service stack that receives data from IoT devices.

### Limitations

Our proof-of-concept solution has been tested for scalability with 4 nodes and works on a secure public network. We tested fail-safeness and replication of data across nodes for the ascertained (mined) data and blockchain. In the future, the architecture can be extended to include a larger number of nodes, and distributed data replication can be tested. However, we are confident that the CHASM architecture will support such scalability as the underlying network and data distribution interfaces are pluggable. Similarly, we believe that our tests performed with >5 devices, including simulators and health apps that track physical activity, can be scaled to a larger number of devices and several types of apps. The PantherChain implementation is device agnostic, and data stored on the blockchain are agnostic to the source of data.

The heterogeneous usability of the blockchain used by providers and device manufacturers, who would now lose control over the data (or would have to purchase data from their customers), to analyze them will lead to reduced times in designing and developing more efficient data. The need for users to share revenues and profits with other users who produce data will be difficult. The risk of losing the private key to access one’s data will make the data completely unusable forever. Therefore, the storage of the private key (or private key generation algorithms) needs to be adhered to closely.

### Conclusions

Overall, we demonstrate the benefits of using blockchain in presenting a unique and novel architecture for mHealth data using multiple devices. Our implementation of a unique blockchain that is configurable with respect to its subcomponents and API layer provides the necessary flexibility and security in addressing privacy, security, and data ownership issues with personal health data. Our findings from implementing the proof of concept with multiple devices and personal data are as follows:

1. With IoT devices and personal mHealth data, blockchains can provide the flexibility of storing, retrieving, and accessing individual user data, despite multiple devices and operating systems generating such data about the individual.
2. Aggregation of data on the individual and providing only individual access to blockchain data using encryption protects the user’s privacy. Using the wallet approach, the user can also transfer or send a copy of his or her data to other wallets through a separate transaction.
3. Our design of mHealth apps on different devices and operating system agnostic apps, which communicate with the blockchain through web services, supports IoT throughput requirements.
4. As stated in the test results above, we demonstrate the performance compliance for IoT devices as well as HIPAA compliance for user data with our design.

Using this implementation, in the future, we could create a *data marketplace* for personalized health care data, where anonymous users can control the generation, quality, and monetization of their health care data, instead of giving data away for free.

## Appendix

Multimedia Appendix 1 Invocation of mobile health data uploaded to PantherChain (Android and Java).

Multimedia Appendix 2 Invocation of mobile health data uploaded to PantherChain (iOS and Swift).

## Abbreviations

API: application programming interface
ASIC: application-specific integrated circuit
CHASM: consensus, hasher, storer, miner
DSRM: Design Science Research Methodology
GDPR: General Data Protection Regulation
HIPAA: Health Insurance Portability and Accountability Act
IoT: internet of things
MD5: Message Digest Version 5
mHealth: mobile health
POW: proof-of-work
REST: representational state transfer
SHA256: Secure Hash Algorithm
