# Supplementary material for: User Control of Personal mHealth Data Using a Mobile Blockchain App: Design Science Perspective
Source: JMIR Mhealth Uhealth. 2022 Jan 20;10(1):e32104. doi: 10.2196/32104 (PMC8814930; doi:10.2196/32104)
Supplement: Multimedia Appendix 1 [file mhealth_v10i1e32104_app1.docx]

Multimedia Appendix 1. Invocation of the mHealth data upload to PantherChain (Android/Java)

| String url = pantherchainbaseurl + "addJson"; final String json = hearrateMonitor.getSummary();  final RequestQueue queue = Volley.newRequestQueue(this); StringRequest postRequest = new StringRequest(Request.Method.POST, url,  response -> { … queue.stop();},  error -> {… queue.stop(); }) {  @Override  protected Map<String, String> getParams()  {  Map<String, String> params = new HashMap<>();  params.put("jsonData", json);  params.put("publicKey", public_key_value);  return params;  } }; queue.add(postRequest); |
| --- |
