# Supplementary material for: User Control of Personal mHealth Data Using a Mobile Blockchain App: Design Science Perspective
Source: JMIR Mhealth Uhealth. 2022 Jan 20;10(1):e32104. doi: 10.2196/32104 (PMC8814930; doi:10.2196/32104)
Supplement: Multimedia Appendix 2 [file mhealth_v10i1e32104_app2.docx]

Multimedia Appendix 2. Invocation of the mHealth data upload to PantherChain (iOS/Swift)

| // create post request  let url = URL(string: "\(self.pantherchainbaseurl)addJson")!  var request = URLRequest(url: url)  request.httpMethod = "POST"  // create post request  var urlComponents = URLComponents()  let q1 = URLQueryItem(name: "publicKey", value: self.public_key_value!)  let data = self.heartRateManager.getSummary().getJsonData()  let q2 = URLQueryItem(name: "jsonData", value: data  urlComponents.queryItems = [q1, q2]  let payload = urlComponents.percentEncodedQuery!.data(using: .utf8)  // submit the request and process results  let task = URLSession.shared.uploadTask(with: request, from:payload) { data, response, error in … }  task.resume() |
| --- |
